# Effect of ureteral stricture in transplant kidney and choice of treatment on long-term graft survival

**DOI:** 10.1007/s11255-023-03669-z

**Published:** 2023-06-12

**Authors:** Jiangwei Zhang, Wujun Xue, Puxun Tian, Jin Zheng, Chenguang Ding, Yang Li, Ying Wang, Xiaoming Ding, Yi Lv

**Affiliations:** 1 Department of Kidney Transplantation, The First Affiliated Hospital of Xi’an Jiaotong University, Xi’an, 710061 China; 2 Department of Hepatobiliary Surgery, The First Affiliated Hospital of Xi’an Jiaotong University, Xi’an, 710061 China

**Keywords:** Kidney transplantation, Ureterostenosis, Open surgery, Luminal surgery, MCA, Survival rate of transplant kidney

## Abstract

**Background:**

To explore the risk factors of ureteral stricture in transplant kidney and the clinical effects of different treatment methods.

**Methods:**

The 62 patients with transplant kidney ureteral stenosis as the experimental group, and another group of recipients from the same donor as the control group (*n* = 59 cases). The risk factors for ureteral stricture and the survival rate of transplant kidney were analyzed and compared. The 62 patients were divided into open operation, luminal operation, and magnetic compression anastomosis (MCA) operation group. The effect of the operation and the survival rate of transplant kidney among the three groups were compared.

**Results:**

In our study, we found that the above differences were statistically significant in clinical data such as gender, multiple donor renal arteries, history of infection, and delayed graft function (DGF) between the two groups (*P* < 0.05). Urinary tract infection and DGF history were the independent risk factors for the development of ureteral stricture. The open operation had the best treatment effect and the survival rate of the transplant kidney, followed by the MCA, the stricture recurrence rate in the luminal operation was the highest.

**Conclusion:**

The ureteral stricture has a negative correlation with the long-term survival rate of the transplant kidney, the curative rate and long-term effect of open surgery are the best, stricture recurrence rate of luminal surgery is high, and it may require multiple operations in the future, the MCA is a new breakthrough and innovation in the treatment of ureteral stricture.

## Introduction

Kidney transplantation is currently the most effective treatment for patients with end-stage renal disease [1]. With the continuous progress and innovation of donor evaluation, organ acquisition and repair, kidney transplantation, ureteral anastomosis and other technologies, the incidence of surgical complications related to kidney transplantation has decreased significantly [2]. However, ureteral stricture in transplant kidney is still one of the most common surgical complications after kidney transplantation at present, and the incidence rate is reported to be 1–8.3% in the literature, which is negatively correlated with long-term graft survival [3–5]. Improper treatment may damage the transplant kidney function and affect the long-term survival rate of the recipient and the transplant kidney [6, 7]. This study systematically reviewed and analyzed the clinical data of 62 patients with ureteral stricture in transplant kidney in the First Affiliated Hospital of Xi’an Jiaotong University from January 2011 to January 2021 and the efficacy of different treatment methods, to provide reference for the clinical diagnosis and treatment process, reduce the incidence of ureteral stricture in transplant kidney, and improve the transplantation effect and survival rate.

## Materials and methods

Sixty-two patients with ureteral stricture in transplant kidney who were admitted to the Department of Kidney Transplantation, the First Affiliated Hospital of Xi’an Jiaotong University from January 2011 to January 2021 were selected as the experimental group. All donor kidneys were obtained from deceased donors (DD). The criteria for inclusion in the experimental group were as follows: a. Patients received kidney transplantation for the first time; b. The ureteral stricture in transplant kidney was definitely diagnosed and surgical treatment was conducted concurrently; c. Patients were followed up in our transplantation center regularly after operation, with good compliance. Exclusion criteria: a. Patients without surgery for ureteral stricture in transplant kidney; b. Loss of follow-up or incomplete information; c. Living donor kidney transplantation; d. The transplant kidney is removed due to surgical complications during the perioperative period; e. Multiple organ transplantation. In order to reduce the effect of donor kidney quality on the transplantation result, the recipients who received the other kidney from the same donor were used as the control group, the recipients in the control group were all patients without transplant kidney ureteral stenosis, 59 cases were selected as the control group (two cases with the transplant kidney removed due to perioperative surgical complications and one case with regular dialysis treatment for oxalate nephropathy recurrence were excluded). All recipients underwent an extravesical tunneled anastomosis of the ureters to establish an anti-reflux mechanism, using a double “J” ureteral stent. The follow-up observation time for patients was 0.5–10 years.

### Immunosuppressive protocol

The immunosuppressive protocol in this study were all formulated in accordance with the diagnostic and therapeutic specifications of the Department of Kidney Transplantation, the First Affiliated Hospital of Xi’an Jiaotong University. The perioperative induction regimens included the application of interleukin-2 receptor antagonist (IL-2Ra, basiliximab) or anti-thymocyte globulin in layers according to the immune risk after renal transplantation. Namely, anti-human T lymphocyte rabbit immunoglobulin (ATG-F) or rabbit anti-human thymocyte immunoglobulin (rATG) was used for immune induction. Maintenance oral immunosuppressive protocol: The basic immunosuppressive protocol initially used in renal transplant recipients was the triple immunosuppressive protocol of calcineurin inhibitor (CNI) + mycophenolate mofetil (MMF) + prednisone (Pred). CNI included tacrolimus (Tac) and cyclosporin A (CsA).

### Clinical manifestations and diagnosis

**Fig. 1:**
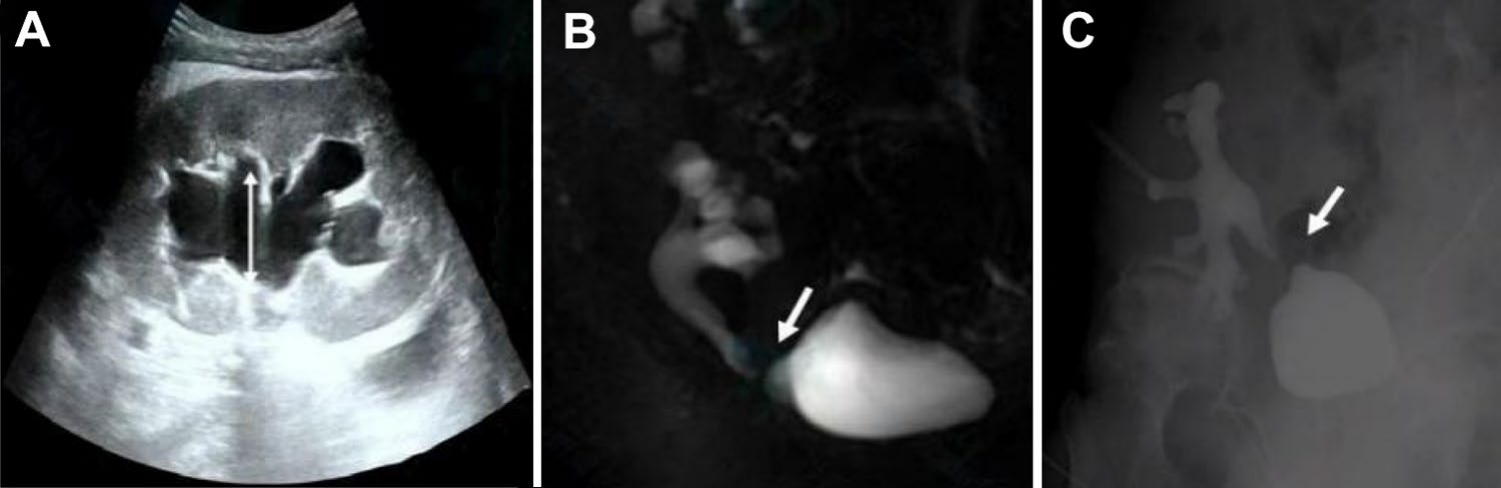
Different diagnostic modalities of ureteral stricture in transplant kidney. **A** The ultrasound showed the transplant kidney hydronephrosis, renal pelvis was separated by approximately 20 mm. **B** MRU can provide three-dimensional images and fully display the shape of stenosis site. **C** Anterograde urography can be performed through the fistula to initially understand the obstruction site and stenosis degree

Early ureteral stricture in transplant kidney mostly occurred within 1–3 days after operation, while late stricture mostly occurred 3 months after operation [8]. Early stenosis was manifested as decreased or no urine in a short time, increased serum creatinine (Scr), distension and pain in the transplant kidney area. Late patients were mainly manifested as mild increase in Scr, and recent hypertension, lower limb edema, or repeated urinary tract infection might occur. The renal pelvis separation was observed by ultrasonography. In a few patients, hydronephrosis (more is < 2 cm) accompanied with mild increase in Scr or normal was found only during routine re-examination. However, complications such as acute or chronic rejection, drug-induced renal injury, transplant kidney artery stenosis, BKV associated nephropathy (BKVAN), and renal disease recurrence should be ruled out, and a transplant kidney puncture biopsy may be performed if necessary. The ultrasonography is the preferred diagnostic method [9], shows the separation of renal pelvis or dilatation of ureter (Fig. 1A), emission computed tomography (ECT) can distinguish functional or mechanical obstruction to a certain extent [8], magnetic resonance urography (MRU) can provide three-dimensional images and fully display the shape of stenosis site (Fig. 1B), it is more suitable for the diagnosis of transplant kidney and ureter stenosis [10].

### Therapeutic regimen

In the selection of treatment for ureteral stricture in transplant kidney, the cause, location and degree of ureteral stricture, onset time, patient’s own will should be fully considered. According to the different treatment modalities, these patients were divided into the open surgery group (*n* = 20), the luminal surgical group (*n* = 27) and the MCA surgery group (*n* = 15). All operations were performed by the same surgical team. Our definition of surgical failure should meet any of the following conditions: a. The operation is terminated due to the failure to relieve the stenosis during the operation; b. The ureteral stent was removed after the operation, and the stricture occurred again; c. The previous operation method needs to be repeated; d: It is converted to other surgical methods.

### Percutaneous nephrostomy drainage

For patients with ureter stenosis in transplant kidney accompanied by decreased urine volume, rapidly increased Scr and inoperable condition within a short period of time, to relieve hydronephrosis and protect transplant kidney function, we suggest that percutaneous nephrostomy drainage should be performed as soon as possible, which is also in preparation for subsequent surgery. Relatively speaking, the anterograde endoscopic operation is simpler and the success rate is higher. In this study, a total of 42 patients underwent percutaneous nephrostomy drainage, including 10 cases in the open operation group, 17 cases in the luminal operation group, and 15 cases in the MCA operation group. After the renal function is normal and the water and electrolyte balance is restored, elective surgery should be performed without infection or active bleeding. If the drainage of the flaccidity-creating tube is poor, normal saline can be used to wash it out. Before the operation, anterograde urography can be performed through the fistula to initially understand the obstruction site and stenosis degree (Fig. 1C).

### Open surgical treatment

Open surgery is still one of the most effective treatments for transplant kidney ureteral stricture. It is suitable for patients with transplant kidney ureteral stricture during the perioperative period, long stricture segment, long stricture time and failure of other treatments [11]. In this study, 20 patients underwent open surgery, including 18 males and 2 females, average age of 50.21 ± 15.30 years old with onset of 8.81 ± 3.42 months. The key to the success of open surgery is the ability to quickly, accurately and atraumatically separate the remaining ureters, so adequate preoperative preparation and surgical planning should be made, and the operations should be performed by the transplant surgeon. The procedures of open surgery were as follows: the skin, subcutaneous tissue and muscular layer were opened layer by layer at 1 cm inside the incision of the original surgery, the lower pole of the transplant kidney was carefully separated, the transplant kidney and ureter were separated under the capsule of the transplant kidney, and the stenotic segment was excised. Intravenous injection of furosemide was given, urine outflow was observed at the ureteral stump. According to the residual length of ureter, we can select end-to-end or end-to-side anastomosis of the transplant kidney ureter and the recipient’s own ureter, or anastomosis of the transplant kidney ureter and ureter-bladder muscle flap, or ureter-bladder re-anastomosis. In this study, the ureter was reconstructed using the Boari flap, and the top of the bladder was exposed, a trapezoidal incision was made to free the 5.0 cm × 1.5 cm mucosal flap of the bladder [12, 13]. The free bladder mucosa was curled, and a 5–0 absorbable suture was made into a tubular shape with a double “J” tube stent placed therein, continuously anastomosed with the end-to-end anastomosis of the transplant kidney ureter and the Boari flap. The 4-0 absorbable suture was used to suture the gap in the bladder wall, and the bladder muscle layer was embedded with silk thread. If the ureteral stump of the transplant kidney is too short, it can also be anastomosed with the end-to-side or end-to-end of the recipient’s own ureter.

### Luminal surgical treatment

**Fig. 2:**
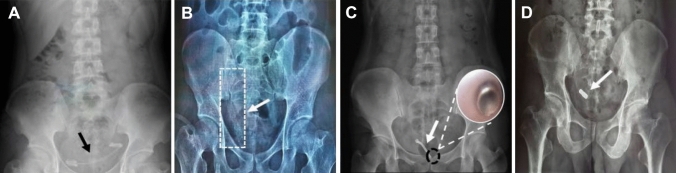
**A** Double “J” tubes placed in patients with transplant kidney ureteral stenosis. **B** Placement of covered metal stents in patients with transplant kidney ureteral stenosis. **C** Placement of a Ni–Ti memory alloy ureteral stent in a patient with transplant kidney ureteral stenosis, the arrow shows memory alloy ureteral stent thermal expansion end. **D** The DM was adsorbed together with the PM

In our study, 27 patients underwent endoscopic surgery for ureteral stricture, 15 males and 5 females, average age of 48.70 ± 15.65 years old with onset of 4.55 ± 1.87 months. For patients with stenosis less than 1 cm and within 6 months after transplantation, priority should be given to luminal surgical treatment. For the ureteral obstruction with percutaneous nephrostomy drainage, the treatment process of intraluminal surgery is basically the same as that of MCA, and ureteroscopic balloon dilatation or ureteral stent placement was performed. If the guidewire cannot pass through the completely occluded ureter, we used an electrocision mirror or laser to cut the ureteral opening, and then place a balloon dilator along the guidewire, balloon diameter 6–10 mm, dilation pressure 10–30 atm, dilation time 3–5 min, and finally place different types of ureteral stents according to the condition, (Fig. 2A–C).

### Magnetic compression anastomosis (MCA) surgical treatment

MCA is a treatment method that uses the attractive force between the anastomosis devices made of magnetic materials to compress the organ wall layer or tissue to cause ischemia and necrosis to recanalize the lumen [14]. MCA is not suitable for all patients due to special factors such as magnets and magnetic field, and the inclusion criteria are as follows: a. The length of transplant kidney ureteral stricture segment ≤ 2 cm, and the appropriate duration of stricture < 3 months; b. Anyone who has signed an informed consent form and is able to cooperate with the treatment and follow-up review; c. Percutaneous nephrostomy must be performed before the operation. Exclusion criteria: a. Patients who are accompanied with severe organ dysfunction, pregnant or lactating women, with mental and psychological disease or those who are in the active infection state; b. Patients with implants (such as cardiac pacemaker, metal denture and fracture fixation plate) interfered with by magnetic field in vivo; c. Magnetic resonance imaging (MRI) examination is necessary or possible in the near future; d. The stenosis segment was too long (> 2 cm), and the attractive force between magnets could not meet the requirement of compression anastomosis; e. The diameter of ureter or urethra is too thin for magnet placement. In this study, the magnetic ring was the third generation rare-earth NdFeB permanent magnet material, which was divided into a daughter magnet (DM) and a parent magnet (PM). Generally, the DM was about 1.5 cm in length and 0.6 cm in diameter, and the PM was about 1.5 cm in length and 0.8 cm in diameter, the dimensions can also be customized according to the specific situation. The procedure of MCA was similar to that of the luminal surgery, in which the DM was inserted anterograde from the nephrostomy tube. Another ureteroscope was inserted into the bladder, and the ureteral scar on the top wall of the bladder was found. The zebra guidewire was threaded into the ureter from the ureteral scar on the top wall through the ureteroscope, and the PM was inserted until the magnets at both ends attracted each other to form a pressing force, and the fistulous tube was re-inserted. After the magnetic ring fell off, two zebra guidewires were inserted anterograde and retrograde respectively, and two F6 double “J” tubes were inserted under the guidewire guidance, and the tubes were retained for at least 3–6 months (Fig. 2D).

In this study, a total of 15 renal transplant recipients underwent MCA surgery. The surgery was uneventful and the magnets were successfully placed. The operations were all completed within 1.5 h, which was significantly shorter than the open surgery time, the bleeding volume was less than 10 ml. The magnetic rings naturally fell off in 14 cases, and the average time was 15.78 ± 2.91 days, one case was taken out through ureteroscope on 72 days. The hydronephrosis was significantly alleviated or disappeared, the double “J” tubes were removed 3–6 months later. Four patients developed fever after surgery, diagnosed as “urinary tract infection”, and improved after anti-infection treatment. Two cases experienced ureteral stricture again after the double “J” tubes were removed, and the double “J” tubes were re-retained for three months, and no stricture occurred in the rest cases. The clinical data of patients in the MCA group are shown in Table 1.

**Table 1:** Surgical data of the 15 cases with ureteral stenosis in transplant kidney treated by MCA

Case	Onset time (months)	Operation time (mins)	Estimated stenosis segment length (cm)	Stenosis area	Time point for magnet ring falling off (days)	Mode for magnetic ring falling off	Postoperative complication
1	11	75	1.5	Anastomotic stoma	72	Endoscopic removal	Urinary tract infection
2	4	55	1.2	Anastomotic stoma	14	Falling off naturally	Urinary tract infection
3	3	90	1.0	Anastomotic stoma	18	Falling off naturally	–
4	2	85	1.0	Anastomotic stoma	21	Falling off naturally	–
5	6	75	1.5	Middle ureter	15	Falling off naturally	Re-ureterostenosis
6	12	105	1.3	Anastomotic stoma	17	Falling off naturally	–
7	7	120	1.6	Lower ureter	13	Falling off naturally	–
8	6	85	1.7	Anastomotic stoma	15	Falling off naturally	Urinary tract infection
9	2	75	1.5	Anastomotic stoma	12	Falling off naturally	–
10	4	65	1.5	Middle ureter	15	Falling off naturally	Urinary tract infection
11	3	105	1.0	Anastomotic stoma	12	Falling off naturally	–
12	11	125	0.8	Anastomotic stoma	14	Falling off naturally	–
13	9	115	1.1	Anastomotic stoma	18	Falling off naturally	Re-ureterostenosis
14	10	120	0.9	Anastomotic stoma	21	Falling off naturally	–
15	6	80	0.7	Anastomotic stoma	16	Falling off naturally	Urinary tract infection

### Statistical methods

SPSS 26.0 and Graphpad Prism 8.0 were used for statistical processing. The quantitative data were expressed as mean standard deviation ($\overline{x} \pm s$). Independent sample t test was applied to test whether the data conformed to the normal distribution or the rank sum test was applied to test whether the data did not conform to the normal distribution. Enumeration data were expressed as case numbers, and the Chi-square test (*χ*^2^) or Fisher’s exact probability test was performed. Kaplan–Meier method was used for survival analysis and survival curve was drawn. The cumulative survival rate at different time points was calculated by life table method.* P* < 0.05 in univariate analysis was included in the Cox proportional risk model to analyze the risk factors of stricture of ureter in transplant kidney. All the statistics in this paper were performed by bilateral test, and the difference between the two tests was statistically significant when the *P* < 0.05.

## Result

### Comparison of general clinical data

There was no statistically significant difference in clinical data such as age, BMI, preoperative Scr, HLA mismatch, perioperative induction protocol, immunosuppression protocol, acute rejection (AR), transplant kidney exploration operation, and pulmonary infection between the two groups (*P* > 0.05). However, for male patients, the donor had multiple renal arteries, a previous history of urinary system infection, CMV infection, BKV infection, and a history of DGF [15, 16], and they were more likely to develop transplant kidney ureteral stenosis, the above differences were statistically significant (*P* < 0.05), as shown in Table 2.

**Table 2:** Demographic characteristics of patients in the ureteral stenosis in transplant kidney group and the control group

Project	Experimental group (*n* = 62)	Control group (*n* = 59)	*t* or *χ*^2^	*P-*value
Age (years)	50.16 ± 10.8	45.67 ± 11.69	0.78	0.48
Gender (male/female)	50/12	38/21	4.02	0.045
BMI	25.18 ± 3.21	22.88 ± 2.64	0.90	0.56
Preoperative SCr (umol/L)	795.14 ± 67.82	729.23 ± 52.96	0.47	0.39
HLA mismatch number	2.56 ± 0.96	2.21 ± 0.85	2.85	2.1
Donor multiple renal arteries%	35.4% (22/62)	18.64% (11/59)	4.32	0.038
Perioperative induction protocol (case)				
IL-2Ra	28	20		
ATG	34	39	1.25	0.26
Immunosuppressive protocol (case)				
Tac + MMF + Pred	40	42		
CsA + MMF + Pred	22	17	0.62	0.43
Complications after kidney transplantation*				
Urinary system infection	25.8% (16/62)	10.2% (6/59)	4.97	0.02
CMV infection	35.5% (18/62)	18.6% (8/59)	4.29	0.03
BKV infection	35.5% (22/62)	18.6% (11/59)	4.32	0.03
AR	8.0% (5/62)	6.7% (4/59)	0.07	0.79
DGF	19.3% (12/62)	6.8% (4/59)	4.12	0.04
Exploration of transplant kidney*	8.1% (5/62)	10.1% (6/59)	0.16	0.67
Pulmonary infection	9.7% (6/62)	13.5% (8/59)	0.44	0.51

*Complications after kidney transplantation refer to the complications occurring from the perioperative period to the diagnosis of ureteral stricture in transplant kidney. Exploration of transplant kidney refers to the secondary operation required due to rupture of transplant kidney, arteriovenous vascular complications, ureteral thrombosis or stenosis during the perioperative period of kidney transplantation

We also calculated the influencing factors of ureteral stricture by Cox proportional risk analysis. The results showed that urinary infection and DGF history were the independent risk factors for ureteral stricture in transplant kidney (*HR* = 2.31, 2.13, *P* < 0.05).

### Comparison of clinical results

**Fig. 3:**
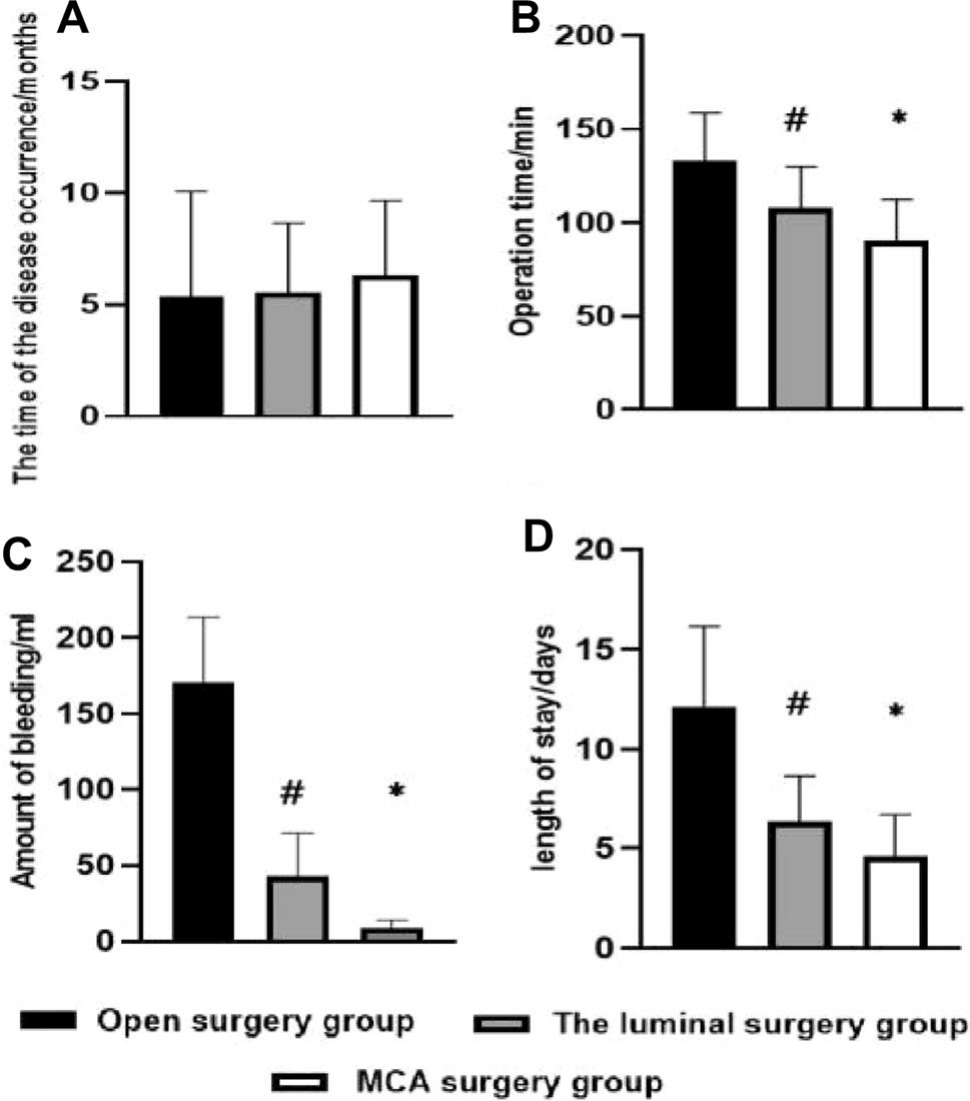
Statistical comparison of clinical data including onset time, operation time, intraoperative bleeding volume and hospital stay of the three surgical treatments. **A** There was no significant difference in onset time among the three groups. **B** Comparison of operation time among the patients in the three groups showed that the differences between the open operation group and the luminal operation group as well as the MCA operation group were statistically significant (*t* = 3.64, 5.29,* P* < 0.01); the differences between the luminal operation group and the MCA operation group were statistically significant (*t* = 2.54, *P* = 0.015). **C** Comparison of intraoperative bleeding volumes among the patients in the three groups showed that the differences between the open operation group and the luminal operation group as well as the MCA group were statistically significant (*t* = 12.87, 14.26,* P* < 0.01); the differences between the luminal operation group and the MCA operation group were statistically significant (*t* = 4.04, *P* < 0.01). **D** As for the comparison of hospital stay among 3 groups, the differences between the open surgery group and the luminal surgery group and the MCA surgery group were statistically significant (*t* = 6.4, 6.84, *P* < 0.01), and the differences between the luminal surgery group and the MCA surgery group were statistically significant (*t* = 2.45, *P* = 0.019)

We compared the onset time, operation time, intraoperative blood loss, and hospital stay of the three surgical treatments (Fig. 3). The onset time of patients in the three groups was (5.36 ± 4.71, 5.53 ± 3.13, and 6.31 ± 3.36 months), respectively, the above differences were not statistically significant. The longest operation time was the open operation group (132.95 ± 25.65 min), and the difference was statistically significant as compared with that in the other two groups (*P* > 0.05), followed by that in the luminal operation group (107.95 ± 25.65 mL), and the shortest operation time was the MCA operation group (90.0 ± 22.01 min). The open surgery group had more trauma and the largest amount of bleeding (183.0 ± 46.80 ml), followed by the luminal surgery group (42.5 ± 28.75 ml), and the MCA surgery group had the least amount of bleeding (12.18 ± 10.64 ml). As for the comparison of hospital stay among the three groups, the open surgery group was affected by the surgical trauma, and the hospital stay was the longest (12.15 ± 3.99 days), followed by the luminal surgery group (6.32 ± 2.29 days), and the MCA surgery group had the shortest (4.63 ± 2.06 days).

### Survival analysis of transplant kidney

**Fig. 4:**
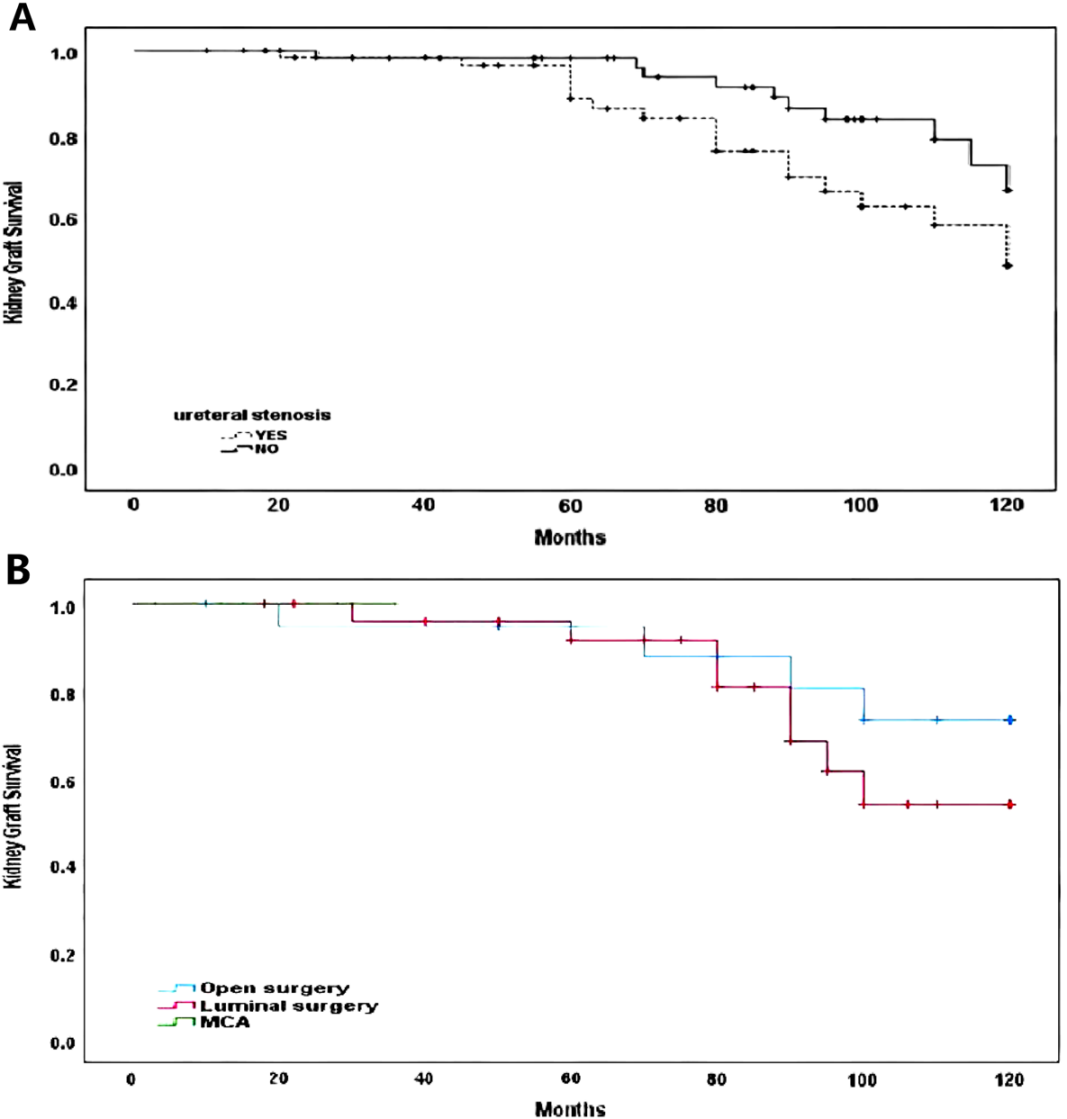
**A** Comparison of transplant kidney survival in the ureteral stricture group and the control group. **B** Comparison of graft survival for three treatment modalities

Graft 10-year survival rate was significantly lower in patients with ureteral stricture in transplant kidney compared with subjects without ureteral strictures (73.3% vs. 53.9%, *P* = 0.038, Fig. 4A). Through the analysis of the survival of transplant kidneys with three different surgical methods, we found that the overall graft survival rate in the open operation group was superior to that in the luminal operation group (*P* = 0.031). As the follow-up time of the MCA group was short (0.5–3 years), there was no case of graft dysfunction, and the longer-term efficacy still needs to be further observed (Fig. 4B).

## Discussion

Ureteral stricture in transplant kidney is a common surgical complication after renal transplantation, which, if treated improperly, can damage the transplant kidney function and affect the long-term survival rate of the recipient and the transplant kidney [17]. Early ureteral stricture in transplant kidney usually occurs 1–3 days after operation and is directly related to operation complications, the main causes include compression of perinephric hematoma, bleeding of renal pelvis and ureter, bending and angulation caused by excessively long ureter retention, over-tightening of tunnel anastomosis of bladder muscular layer, compression of spermatic cord (the one with spermatic cord retention), etc. [18]. Early obstruction generally requires open surgery, of course, most of ureteral stricture in transplant kidney occurs at 3–6 months after surgery [19], which is the difficulty and focus of the current treatment, pathogenesis such as ureteral ischemic necrosis, ureteral stones, scar constitution lead to fibrosis around the ureter, chronic urinary system infection, AR [20], DGF [21], CMV, BKV infection [22], etc., of which ureteral ischemic necrosis is the most common cause of stenosis and obstruction, mostly in the ureter and bladder anastomosis [23].

**Fig. 5:**
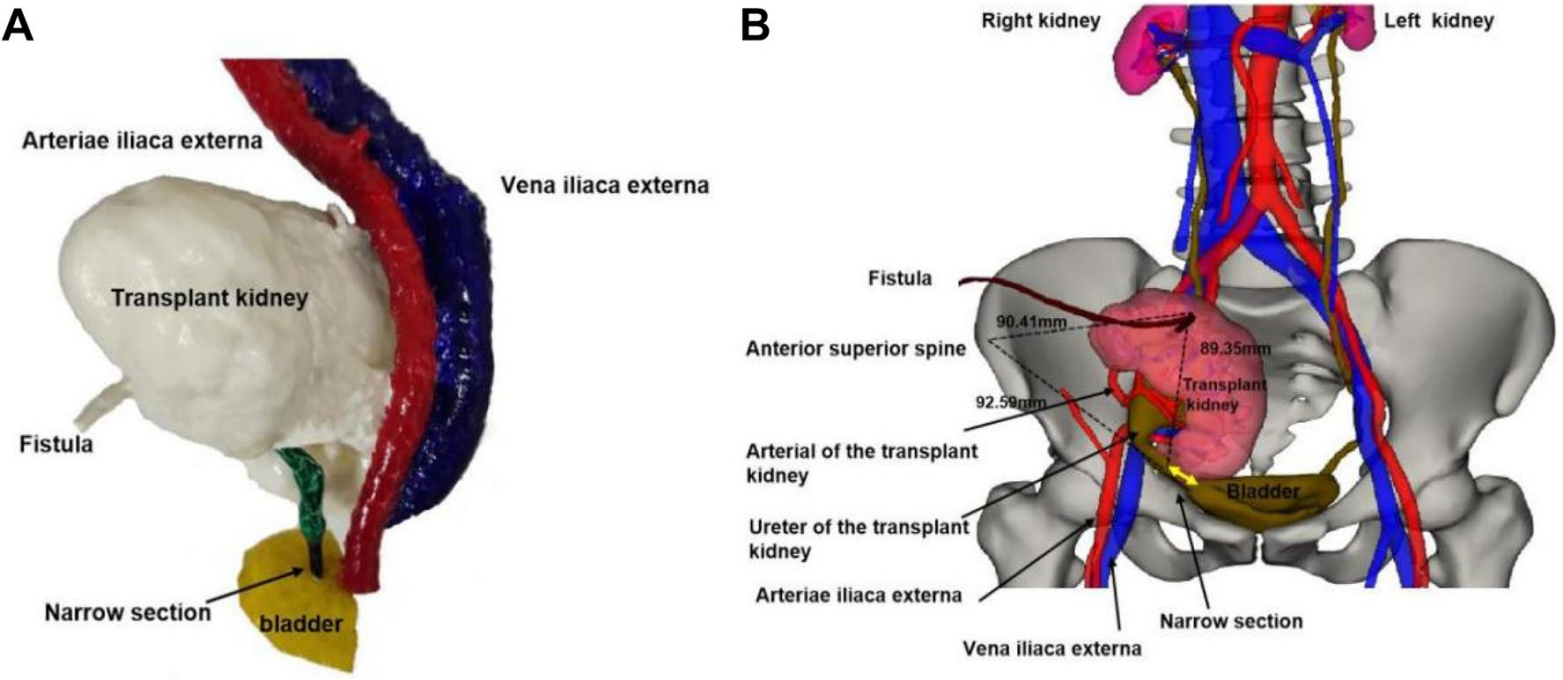
Using three-dimensional reconstruction and 3D printing technology, we can integrate the tomographic images on the two-dimensional plane into a three-dimensional form, which helps surgeons to construct a three-dimensional thinking, displaying the anatomical structure of the surgical site, the orientation of the surrounding tissues, blood vessels and organs, and their adjacent adhesion from multiple angles, and formulating a surgical plan or performing a simulation operation according to the model, thereby reducing the operation risk and improving the operation success rate. **A** 3D printed physical model of ureteral stenosis in transplant kidney can be used to visually observe and feel the anatomical structure from multiple angles. **B** A three-dimensional reconstruction model of ureteral stenosis in transplant kidney can be used to create the “three-point positioning method” (dotted triangle in the figure) with the anterior superior iliac spine, fistula, and ureteral stricture in transplant kidney as the fulcrums to find the location of the ureteral stump, and measure the distances to be 90.41 mm, 92.59 mm, and 89.35 mm, respectively, to guide the operation according to the measured results

The methods of treatment for ureteral stricture in transplant kidney in this study were divided into open surgery, luminal surgery, and MCA surgery. Each treatment has its own advantages and disadvantages, as the corresponding treatment will be selected according to the different conditions and patients’ wishes. We recommend the placement of a ureteral stent regardless of the surgical procedure, which is beneficial to the recovery of the ureteral anastomosis. Some studies have shown that the placement of a stent through the ureteral-vesical anastomosis is an independent protective factor against ureteral complications after kidney transplantation [24, 25]. Open surgery mainly has the problems of unclear anatomical layers, severe tissue adhesion, large trauma, much bleeding, and long operation time, especially for patients with a long time after transplantation and a history of infection, which may result in the failure of the operation due to the fact that the transplant kidney and ureter cannot be accurately located and separated, or the ureter is damaged or the length of the ureter is insufficient. We successfully isolated the ureter using urography, intraoperative methylene blue staining and imaging, three-dimensional reconstruction, and 3D printing, and completed the secondary anastomosis of the transplant kidney and ureter, as shown in Fig. 5.

Although open surgery is more invasive than other surgical procedures, it is usually considered as the “gold standard” for the treatment of ureteral stricture in transplant kidney [26]. Faenza considered open surgery to be the best treatment option for ureteral stricture in transplant kidney caused by surgical factors during the perioperative period [27]. The open operation has an open operation field, so that the anatomical relationship between the stenotic site and the surrounding tissues can be understood more clearly, the etiology can be clarified, and the problem of ureteral stenosis can be thoroughly solved from the source, so that the patient is not easy to relapse, and the ureteral stent does not need to be retained or replaced for a long time, so that the long-term effect is exact, and the open operation treatment is still feasible for cases in which other treatment methods fail [28]. In this study, 20 patients underwent open surgery, and 19 patients underwent successful surgery, one patient developed secondary stenosis one month after surgery and switched to MCA treatment, with the success rate of surgery reaching 95.0%. Among them, 17 cases experienced the stricture of transplant kidney ureter in a long term, and 3 cases experienced it during the perioperative period. Eleven cases underwent ureterovesical anastomosis, one case underwent end-to-side anastomosis of transplant kidney ureter and own ureter, and eight cases underwent ureter reconstruction with Boari flap. Except for one case of secondary stenosis, the other patients experienced no postoperative stenosis, urine leakage, urinary system infection, massive hemorrhage and other related complications, and the overall therapeutic effect was good.

Luminal surgery includes balloon dilatation, electrocision of the stenotic segment, and stent placement, and can be treated by single or multiple methods in combination. We do not advocate simple balloon dilatation treatment. The recurrence rate of stricture after balloon dilatation treatment of transplant kidney ureteral stenosis is high up to 50%, and its application scope is narrow [29]. Reus believed that the balloon dilatation with the stenosis time within 3–6 months or the stenosis segment within 2 cm was ideal, while the treatment with the stenosis duration > 6 months or the stenosis segment > 2 cm was less effective [30]. Endoscopic ureterotomy in the stenotic segment was performed, in which the fiber ring in the stenotic segment was cut, and the surrounding proliferative tissues were removed together, with good clinical effect. However, most of the ureters of the transplant kidney are anastomosed to the “ceiling” at the top of the bladder, and the anastomotic direction of the tunnel in the transplant kidney and ureter makes an excessive angle with the internal orifice of urethra, and most of the stenotic sites are located at the anastomoses, which makes it difficult for the ureteroscope to reversely find the stenotic position of the anastomoses. In particular, the stricture is caused by factors such as excessively long stenotic segment of ureter, tortuosity, discount, and peritubular compression, and the success rate of luminal surgery treatment is not high. The operator is required to have a good endoscopic operation technique. After repeated multi-angle attempts, it is very likely to damage the transplant kidney and ureter. In this study different types of ureteral stents were placed according to the situation, such as double “J” tubes, covered metal stents, and memory alloy stents. 27 patients underwent endoluminal surgery, during the follow-up period, 7 patients experienced restenosis, 6 patients underwent stent replacement surgery, and 1 patient underwent the MCA treatment. The success rate of surgery was 74.07% (7/27), and the overall therapeutic effect was modest.

In recent years, the magnetic surgery technology has developed rapidly and been widely used, including MCA, magnetic anchoring technology, magnetic navigation technology, magnetic levitation technology, magnetic tracer technology, etc. [31–33]. MCA utilizes the “non-contact” magnetic field force characteristics of magnets to persistently compress the target tissue clamped therein in a “non-penetrating” mode, resulting in ischemia-necrosis, to achieve the purpose of recanalization [34]. At present, MCA has been initially applied in biliary tract, gastrointestinal tract and vascular anastomosis, with significant clinical effect [35–37]. Our transplant center has reported the treatment of ureteral stricture in transplant kidney with MCA for the first time in China, proving the safety and feasibility of this treatment, which is a new attempt for disease treatment in the field of kidney transplantation and also provides methods and ideas for the treatment of other diseases. The MCA technique is similar to luminal surgical treatment in terms of surgical procedures, but it has more stringent requirements for applicable populations. We suggest that the length of the ureteral stricture should be ≤ 2 cm, and the appropriate duration of stricture should be less than 3 months. The recanalization time is mainly related to the magnitude of the magnetic force between the magnets, the length and nature of the pressing part, etc. The distance between the magnets becomes smaller and smaller with the passage of time after the PM and DM attract each other. Therefore, the magnet position and urine volume should be closely monitored during the recanalization time. Patients who have undergone percutaneous nephrostomy are often accompanied by the symptoms that the spontaneous urination volume through the original urethra is significantly reduced or even disappeared. If the spontaneous urination volume increases after MCA surgery or returns to normal, it indicates the possibility of magnetic ring recanalization and dropping out (some female patients can directly discharge the magnet out of the body), as a result, the patient should seek medical treatment as soon as possible. In this study, 15 renal transplant recipients underwent MCA surgery. Among them, 14 magnetic rings naturally fell off, with the average time of 15.78 ± 2.91 days, and one case was taken out through ureteroscope on 72 days. The reason was that the patient underwent open surgery, and the scar tissue between magnets was long and hard. Except for two cases where the ureteral stricture occurred again after the double “J” tubes were removed, and the double “J” tubes were re-retained for more than 6 months, no stricture occurred in the rest cases, and the success rate of surgery was 86.67%.

Compared with the control group, the clinical data such as age, body mass index, preoperative blood creatinine, HLA mismatch number, perioperative induction protocol, immunosuppressive protocol, AR, transplant kidney exploration, and pulmonary infection in the group with transplant kidney ureteral stenosis were not statistically significant (*P* > 0.05). Our study confirmed that male patients were more likely to suffer from ureteral stricture in transplant kidney, which might be related to the anatomical structure of male urinary system. The previous infection history of urinary system, CMV, BKV and other diseases may cause inflammatory hyperplasia and interstitial fibrosis of ureter. In addition, DGF leads more prone to ureteral strictures, the mechanism is not yet clear. These factors ultimately increased the incidence of ureteral stricture in the transplant kidney, and the above differences were statistically significant as compared with those in the control group (*P* < 0.05). Ureteral stricture in transplant kidney was affected by a variety of factors. The results of Cox proportional risk model analysis showed that urinary system infection and DGF history were the independent risk factors for ureteral stricture in transplant kidney (*HR* = 2.31, 2.13, *P* < 0.05). We compared the data of onset time, operation time, intraoperative bleeding volume and hospital stay of the three surgical methods. There was no statistical difference in the onset time of patients in the three groups, and most of them occurred in 3–6 months after operation. The open group had the longest operation time, the largest amount of bleeding, and the longest hospital stay, followed by the luminal group, and the MCA group had the smallest surgical trauma. Graft survival was significantly lower in patients with ureteral stricture in transplant kidney compared with subjects without ureteral strictures (73.3% vs. 53.9%, *P* = 0.038). We confirmed that the graft survival time of patients with transplant kidney ureteral stenosis treated by open surgery was superior to that of luminal surgery, and the mechanism is still unclear. In view of the fact that the luminal surgery group was more likely to suffer from secondary stenosis or the double “J” tubes need to be replaced several times in subsequent operations, resulting in repeated kidney injuries. Besides, a higher incidence of urinary tract infections or other complications may empirically reduce immunosuppressive drugs to control infection, increase the incidence of chronic rejection, and ultimately may affect graft function. Due to the short follow-up (0.5–3 years) in the MCA group, there were no cases of graft failure.

It has been proved in our study that ureteral stricture in transplant kidney is negatively correlated with the long-term survival rate of the graft, and it should be effectively prevented from occurring on the basis of risk factors. The surgeon’s most important method to prevent and reduce the occurrence of ureteral stenosis is to improve the surgical precision. In the absence of technical problems, ureteral ischemia is considered to be the main cause, because the only artery supply of transplant kidney and ureter comes from the main renal artery or its lower pole branch. The lower pole accessory artery of kidney and the adipose tissue around ureter should be preserved as much as possible to protect the blood supply of ureter. In addition, active prevention and treatment of DGF, viral infection, urinary infection, calculi after renal transplantation are also the focuses of reducing the occurrence of ureteral stricture. At present, there are many treatment methods for ureteral stricture in transplant kidney, with their own advantages and disadvantages. The curative rate and long-term effect of open surgery are the best, stricture recurrence rate of luminal surgery is high, and multiple operations may be required in the follow-up, MCA is a new method for the treatment of transplant kidney ureteral stricture, which enriches the clinical treatment methods and has a safe and reliable effect. However, considering that the sample size of this study is still small, and the observation and follow-up period of some cases is short, there may be restenosis, anastomotic leakage, urinary system infection, and reflux pyelonephritis, and the longer-term efficacy still needs further observation.

## Data Availability

The raw data supporting the conclusion of this article will be made available by the authors, without undue reservation.
